# Spatiotemporal evolution and the multidimensional proximity mechanism of megaproject innovation networks

**DOI:** 10.1371/journal.pone.0322630

**Published:** 2025-05-23

**Authors:** Haiyan He, Luting Xu, Yijing Huang

**Affiliations:** School of Architecture and Civil Engineering, Chengdu University, Chengdu, China; University of the West of England, UNITED KINGDOM OF GREAT BRITAIN AND NORTHERN IRELAND

## Abstract

Megaprojects necessitate collaborative innovation that transcends organizational, departmental, industrial, and regional boundaries, culminating in the formation of innovation networks. Utilizing data from the megaprojects awarded in the 1st to 19th Zhan Tianyou Awards as a foundation, this study employed social network analysis, Ucinet, and ArcGIS to construct both the collaborative innovation network of participating units in megaprojects and the collaborative innovation network of cities involved in megaprojects. The study analyzed the spatiotemporal evolution characteristics and core organizations within the collaborative innovation network of participating units in megaprojects, and discussed the core-periphery structure and spatiotemporal evolution of the collaborative innovation network among cities engaged in megaprojects. ArcGIS and Spass software were employed, along with a negative binomial gravity model, to investigate the role of multidimensional proximity in the construction of collaborative innovation networks among cities for the 1st-5th, 6th-10th, 11th-15th, and 16th-19th sessions of megaprojects. The key conclusions were as follows. (1) Participating units of the railway system occupy core positions in the collaborative innovation networks of participating units and hold absolute ‘power’ in the networks. (2) Nuclear cities in the city collaborative innovation networks appear to transfer from the eastern first-tier cities, Guangzhou and Shenzhen, to the eastern second-tier cities, Taiyuan, Jinan, Hefei, and the central-western second-tier city, Chengdu. (3) Geographical proximity, institutional proximity, cognitive proximity and social proximity all promote cities’ collaborative innovation in megaprojects. This study still has some limitations. First, this study only uses a single measure of proximity and does not measure it from multiple perspectives. Second, this study used the number of megaproject collaborations as the dependent variable, without considering the relationship between network structure and actual innovation outcomes or project performance.

## 1 Introduction

It is estimated that by 2030, 5 billion people worldwide will be living in cities [1], creating a widespread demand for mega infrastructure projects. Mega infrastructure projects, hereinafter referred to as megaprojects, are large-scale public projects with huge investment scales, long implementation periods, extremely complex technologies, and far-reaching impacts on society, the economy and the ecological environment [2,3]. A performance paradox exists in megaprojects [4]. On the one hand, the number and scale of megaprojects are increasing globally. According to McKinsey, the entire world will invest $57 trillion in infrastructure investment, mostly for megaprojects. Economists call it “the largest investment growth period in human history” [5]. On the other hand, the economic, environmental and public support performances of these megaprojects are often surprisingly poor [4]. Taking economic performance as an example, megaprojects generally present problems of cost overruns and income insufficiency [6,7]. Flyvbjerg’s investigation of 258 projects, including railways, sea bridges and highways, shows that 40% cost overruns of megaprojects are very widespread, and 80% cost overruns of railway projects are not uncommon [6]. In addition, income insufficiency has seriously affected the economic performance of megaprojects. For instance, the Channel Tunnel, opened in 1994, achieved 6.9 million passengers after six years of operation, which was only 43% of the predicted volume [4], and the actual traffic volume in its opening year of the Paris high-speed railway in France was only 25% of the predicted volume [4].

Innovation is a critical factor in the performance enhancement of megaprojects [8]. Due to technological complexity, deep uncertainty and knowledge integration [9], megaprojects require collaborative innovation across organizations, departments, industries and regions [10]. Although engineering or projects possess one-time characteristics and organizational cooperation is temporary [11], from the perspective of greater market cooperation, cooperation among some enterprises may be long-term and sustainable [12,13], and the cooperation network of a single megaproject is merely part of a larger market cooperation network or a corporate strategic cooperation network [14–16]. Megaproject innovation is not isolated; across a single megaproject, innovation clusters, innovation networks [8] and innovation ecosystems [3] have gradually been generated.

Existing studies have emphasized the significant role of collaborative innovation in megaproject construction, proposed project-based innovation clusters and cross-organizational innovation networks, and analyzed the evolutionary and topological characteristics of innovation networks. These studies put forward valuable conclusions from different perspectives and showed that megaproject innovation is still the hotspot and priority of current research. However, there is still a gap in the existing research on the network structures, spatiotemporal evolution characteristics and multidimensional proximity mechanism of the city collaborative network of megaprojects, which affects the enrichment and development of the innovation theory of megaprojects and is not conducive to improving the innovation efficiency of megaprojects. Therefore, based on the data on megaprojects awarded by the 1–19th sessions Zhan-Tianyou Prize (ZTP), this research has the overarching aim of describing the network characteristics and spatiotemporal evolution of the collaborative innovation networks of megaprojects based on methods and tools, including social network analysis (SNA), Ucinet and ArcGIS, and exploring the multidimensional proximity mechanism of cities’ collaborative innovation networks of megaprojects by a negative binomial model to provide scientific guidance for leveraging the advantages of proximity to enhance the collaborative innovation performance of megaprojects and for adjusting the appropriate degree of innovation openness to fully exploit the advantages of proximity.

## 2 Literature review

### 2.1 Collaborative innovation in megaprojects

The fundamental purpose of megaproject innovations is to solve the organizational or technical problems continually occurring during construction and handover processes. Innovation subjects of megaproject technology innovation, unlike common enterprise technology innovation, are mutually nested and dynamically altered in different stages of the whole life cycle (concept, approval, design, construction, operation, etc.) [10,17]. Hence, a series of collaboration mechanisms, including organizational communication processes, supply chain management, and incentive measures, should be established [14,18]. In view of the importance of collaboration to megaproject innovation, researchers have mainly constructed collaborative governance mechanisms from two aspects: process and organization. Aiming at the problem of collaborative innovation of megaprojects between contractors and subcontractors under the engineering procurement construction mode, Zhu et al. [19] discussed the impact of spillover effects, income distributions, incentives and subsidies on evolutionary stability strategies in collaborative innovation. In terms of organizations, recent studies suggest that cross-organizational cooperation in megaprojects involves complex networks [3,5,19]. Based on data from more than 200 architecture, engineering, construction (AEC) projects that won the State Science and Technology Advancement Prize of China from 2000 to 2014, Han et al. [8] established the AEC collaborative innovation network and found that universities and colleges have a significantly higher betweenness centrality than other innovation subjects and are key network nodes that promote innovation diffusion and the integration of theory with practice.

### 2.2 Megaproject and social network analysis

The SNA method is a foundational tool and research paradigm for quantifying individual or organizational relationships and information diffusion [8] and is extensively utilized in stakeholder management, risk management and collaborative innovation of megaprojects and complex projects. From the perspective of stakeholder management, Yuan et al. [20] established a stakeholder network and a social risk factor network to explore the impact of a complex environment, irrational behavior of individuals or organizations, and diversified interaction between the environment and behavior on the social risk of engineering projects in high-density urban areas. Wang et al. [21] constructed a cross-organizational network of owners and stakeholders to analyze the different roles and linkages of stakeholders in water conservancy and hydropower projects during the project feasibility study, implementation, procurement and handover phases. Son and Rojas [22] introduced agent-based simulated collaborative evolution into the interorganizational network of construction project teams.

From the perspective of collaborative innovation, Liu et al. [23] constructed collaboration networks of intercontractors’ projects that won the National Quality Award Projects of China and provided a comprehensive and systematic picture of the principle of how interorganizational networks evolved from an industry perspective. Han et al. [8] collected data on more than 200 projects over a 15-year period that were awarded the State Science and Technology Advancement Prize of China, examined each of these innovative collaboration cases, and used SNA techniques to understand the network characteristics and to examine the evolutionary patterns over time. Zeng et al. [3] discussed the evolution of the patent relationship network of the Hong Kong-Zhuhai-Macao Bridge. This shows that due to the greater complexity, stronger dynamics, higher obvious diversity and network heterogeneity of megaproject innovation networks, innovation entities from different departments, industries and regions need to participate in innovation activities.

## 3 Methods

### 3.1 Research hypothesis

This paper synthesizes the research of Table 1, taking into account the dimensions of geographical, cognitive, institutional, and social proximity, and combines the characteristics of Chinese city attributes to quantitatively analyze the impact of multidimensional proximity on the structure of collaborative innovation networks of cities in megaprojects.

**Table 1:** Studies on the impact of multidimensional proximity on innovation networks.

Authors	Research Subject	Multidimensional Proximity	Conclusion
Du et al. (2023) [24]	City-cluster innovation network	Institutional proximity, cognitive proximity, technological proximity	The effect of multidimensional proximity on innovation link strength shows typical city-cluster heterogeneity
Liu et al. (2024) [25]	New energy vehicle industry innovation cooperation network	Geographic proximity, technological proximity, organizational proximity	Geographic and technological proximity have a significant positive impact on cooperative innovation performance, while organizational proximity has a significant negative impact
Cao et al. (2022) [26]	Medical research institution cooperation network	Geographic proximity, institutional proximity, social proximity, cognitive proximity, and cultural proximity	Geographic, institutional, social, and cognitive proximity promote the formation of the regional medical innovation cooperation network, while cultural proximity has no significant effect
Zhang et al. (2024) [27]	Synthetic industry global innovation network	Geographic proximity, institutional proximity, technological proximity, cultural proximity, and social proximity	Geographic, technological, and institutional proximity’s promotion of cross-border technological innovation cooperation in energy-saving and emission-reduction industries keeps strengthening. Social proximity’s positive effect remains high in all three stages, and cultural proximity’s impact is consistently insignificant

#### 3.1.1 Influence of geographic proximity on collaborative innovation networks.

Geographical proximity, also known as spatial or regional proximity, refers to the degree of spatial distance between innovative entities [25]. It is the most important and widely used proximity indicator and is significantly associated with collaborative innovation, meaning that such innovation largely occurs among geographically proximate innovators, organizations, and regions. Firstly, geographical proximity facilitates face-to-face communication among innovators, promoting the transfer of knowledge and technology. Secondly, the coordination of collaborative innovation requires communication and spatial mobility of technical personnel; geographical proximity helps to effectively reduce the time and economic costs associated with the movement of people and materials in the collaborative innovation process. Thirdly, the knowledge externality effect is stronger the smaller the spatial distance, leading to higher spillover efficiency, which promotes the smooth transfer of tacit knowledge and drives the success of collaborative innovation. Fourthly, geographical proximity reduces opportunism risk, researchers argued that within geographically proximate areas, instances of organizational opportunism spread quickly, hindering the establishment of new connections with other organizations, and organizations tend to adopt more reciprocal strategies in their cooperation. Fifthly, when selecting cooperation partners, innovators need to gather certain information, such as operational status and reputation; collaborating with geographically proximate organizations can effectively reduce the cost of information search [25].

Megaproject innovations are characterized by temporary cross-layer organizational structures [28] and exhibit complexities in the integration of organizational structures and engineering technologies [29–31]. These require the establishment of tight innovation networks among stakeholders, including owners, designers, and contractors, to overcome challenges [14]. They also necessitate face-to-face negotiations and offline agreement signings for innovation cooperation, technology transfer, and commercialization of outcomes. Geographic proximity can enhance communication among participating units and facilitate the spread of tacit knowledge, as well as reduce the spatial and temporal costs for these units in the processes of contract signing, execution, and modification. Therefore, this paper proposes the following hypothesis.

H1: Geographic proximity has a positive effect on city collaborative innovation networks of megaprojects.

#### 3.1.2 Influence of institutional proximity on collaborative innovation networks.

Institutional proximity refers to the similarity in the informal institutional constraints (social norms, cultural values) and formal rules (policies, laws, and regulations) that govern participants. Institutional similarity can reduce uncertainty in cooperation and lower transaction costs [32]. Institutional proximity is influenced by the overall urban planning and development strategies. Similar institutional frameworks facilitate the spread of technical knowledge among cooperating entities, providing a set of shared standard procedures and mechanisms that enable rapid establishment of collaborative and competitive relationships. This smoothens the flow of resources, aids in forming stable expectations among interacting entities, and more effectively promotes collaborative innovation outcomes and the commercialization of results.

Chinese cities operate under a strict administrative hierarchy, and urban policies reflect this tiered characteristic. Cities with higher administrative ranks tend to have greater marketization in engineering innovation, richer social capital, and more favorable industrial policies. In the realm of megaprojects, governments select participating units through a bidding system. The bidding system for megaprojects is characterized by administrative grading, with the central government’s *Law of the People’s Republic of China on Tendering and Bidding*, provincial government regulations on tendering and bidding management, and implementation rules issued by provincial capitals and prefecture-level cities. Differences in standards and systems increase the difficulty of innovation in megaprojects [28]. Institutional proximity, which indicates a high degree of similarity in relevant systems, is conducive to reducing cooperation uncertainties and eliminating implicit barriers [33]. Therefore, this study proposes the following hypothesis.

H2: Institutional proximity positively influences collaborative innovation networks of megaprojects.

#### 3.1.3 Influence of cognitive proximity on collaborative innovation networks.

Cognitive proximity captures the alignment in cognitive understanding and the similarity in the distribution of knowledge endowments among collaborating entities [34]. It is influenced by non-institutional and non-policy factors, reflecting the natural and subjective cognitive similarities among collaborators, which are constrained by spatial factors and natural elements such as cultural dialects and kinship ties, thus exhibiting a strong embeddedness characteristic. Cognitive proximity aids in the interaction and communication between entities; an appropriate level of cognitive proximity can foster mutual understanding of the same subject matter and enhance the transfer and transformation of knowledge. When both collaborators share a similar cultural environment and common social norms and values, barriers to communication are reduced, barriers to social trust are lowered, and the potential for cooperation is increased [34].

Megaproject innovations are not only subject to external environments such as standards, systems, and qualifications but also rely on the dissemination of tacit knowledge and collaborative communication among innovators. Similarities in cultural environments and understandings of cultural values can bring a sense of identity and cultural belonging within the same cultural zone, while also reducing information barriers between innovators, making the spatial flow of knowledge, information, and capital more fluid [34], thereby promoting collaborative innovation in major projects. Thus, this paper proposes the following hypothesis.

H3: Cognitive proximity has a positive effect on collaborative innovation networks of megaprojects.

#### 3.1.4 Influence of social proximity on collaborative innovation networks.

Social proximity refers to the extent to which actors belong to a shared relational space, manifested in the closeness of various relationships within social networks. Research by Boschma et al. suggested that social relationships built on friendship, trust, and a history of cooperation act as catalysts for knowledge spillovers, especially informal communication, which greatly facilitates the spread and learning of tacit knowledge essential for innovation. Additionally, innovators tend to establish connections with friends of friends; such indirect relationships through “intermediaries” are superior to general anonymous relationships and help form stable triadic closure structures. This reduces cooperation uncertainty and the distortion and loss of information in interaction, enhancing the efficiency of technical collaboration [35]. On the other hand, it strengthens dense interactions between actors, curbs opportunism, and avoids potential risks.

Megaproject innovations require substantial inputs of knowledge, capital, and labor at the initial stage, with a highly complex and uncertain innovation process, and the transformation of innovation outcomes is also clearly defined. A good foundation of social relationships and mutual trust can significantly reduce cooperation costs and transaction risks, thereby increasing the likelihood of establishing partnerships. Social proximity indicates the degree of overlap between two urban partners; the higher the social proximity index, the greater the number of common collaborators between two cities, and the higher their cooperation intensity, meaning cities are more willing to establish cooperative relationships with the collaborators of their partners. Therefore, this study proposes the following hypothesis.

H4: Social proximity has a positive effect on collaborative innovation networks of megaprojects.

### 3.2 Data sources and processing

#### 3.1.1 Data sources.

The data for this study were derived from the award-winning projects of the Zhan Tianyou Prize (ZTP) in Civil Engineering in China. Established in 1999 by the China Civil Engineering Society and the Beijing Zhan Tianyou Civil Engineering Science and Technology Development Foundation, the ZTP is a scientific and technological award recognized by the state, accredited by the Ministry of Construction, and approved by the Ministry of Science and Technology. It honors significant engineering projects that demonstrate notable achievements in scientific and technological innovation and the application of new technologies [36]. The ZTP has become the premier award for scientific and technological innovation in the field of civil engineering construction in China, representing the highest level of expertise in the engineering field [36].

#### 3.1.2 Data processing.

The ZTP had been selected annually and publicly announced by the China Civil Engineering Society on its official website. As of the end of 2022, the ZTP had been awarded for 19 sessions, with a total of 549 projects receiving the honor. The data processing for this study is as follows. Initially, raw data were obtained from the official website of the China Civil Engineering Society, including the award year, project name, and main participating units. Subsequently, the names of the award-winning enterprises or organizations were extracted and organized, resulting in a total of 1,719 enterprises or organizations being recognized. Finally, the registration or residential locations of the award-winning enterprises or organizations were identified through Tianyancha searches to determine the cities in which they are based.

### 3.3 Network construction

Referring to the studies of Han et al. [8], Liu et al. [23] and Li et al. [36], this study constructed collaborative innovation networks based on the data of megaprojects awarded by the ZTP. Collaborative innovation networks involve engineering nodes, organization nodes and location nodes and the relationships between these nodes. Among them, the engineering nodes are the winning projects, the organization nodes are the winning enterprises or organizations, and the location nodes are the cities where the winning enterprises or organizations are located. To discuss the spatiotemporal evolution of the networks and their multidimensional proximity mechanism, this study divided the megaproject innovation networks into the following two categories. The networks formulated by the winning enterprises or organizations as nodes are the CINPU of megaprojects, and the networks formed by the cities where the winning enterprises or organizations are located are the CCIN of megaprojects. With the help of UCINET and ArcGIS, network visualization, network structure analysis and spatiotemporal evolutionary characteristics were summarized.

#### 3.2.1 Degree centrality.

Degree centrality is an indicator of the extent to which a network node is connected to other nodes in the network, reflecting the degree to which the node is close to the central hub position in the network and the degree of acquisition and control of resources. The higher the degree centrality of the participating units in the collaborative innovation network of megaprojects, the more direct links formed with other participating units through collaborative innovation activities, and the richer the knowledge and information resources obtained.

Based on the research of Liang et al. [37], the calculation of the degree centrality $DC_{m}$ of the participating unit m is shown in Eq. (1). Among them, k is other participating units except participating unit m, and $X_{mk}$ represents the connection between the participating units when the two participating units jointly participate in at least one megaproject $X_{mk}=1$; otherwise, $X_{mk}=0$.


$$DC_{m}=\sum_{k}X_{mk}\backslash )$$ (1)

#### 3.2.2 Betweenness centrality.

Freeman and Newman proposed the calculation technology of node betweenness centrality. Based on the existing research, this study adopts Eq. (2) to calculate Freeman betweenness centrality. where $g_{mk}$ is the number of shortcuts that must be passed between participating unit m and participating unit k_,_ and $g_{mk}(m)$ is the number of participating units m in the shortcut path of participating units m and participating units k.


$$BC_{m}=\sum_{m<k}g_{mk}(m)/g_{mk}\backslash )$$ (2)

#### 3.2.3 Core-Periphery detection.

Different cities in the CCIN are located in different network positions, as are their importance in the networks. Core-periphery detection, according to the closeness of the nodes in the CCIN, divides the nodes in the network into three layers: the core layer, the semicore layer, the semiperiphery layer, and the periphery layer. Through core-periphery detection, the cities located at the core positions and the cities located at the periphery positions in the CCIN can be clarified. Referring to the research of Liang et al. [37], this study constructed a continuous core-periphery model to calculate the core degrees of each city node, as shown in Eqs. (3) and (4).


$$\rho =\sum_{m,k}X_{mk}\delta_{mk}\backslash )$$ (3)

$$\delta_{mk}=c_{m}c_{k}$$ (4)

where ρ is the measure value; when ρ reaches the maximum, the network presents a core-periphery structure; $\delta_{mk}$ is the matrix under the ideal state; $c_{m}$ and $c_{k}$ are the core degrees of node m and node k, respectively, and both are nonnegative vectors [37]. The calculation of the core degree was realized by the core/periphery module in UCINET.

### 3.4 Negative binomial regression

#### 3.4.1 Variable measurements.

(1) Geographical proximity. Geographical proximity represents the spatial distance between cities in the CCIN. Relevant research indicates that short-distance geospatial space reduces transaction costs and provides face-to-face communication opportunities, thereby promoting knowledge spillovers, especially the dissemination of tacit knowledge. Referring to [37], this study adopted the standardized actual distance to measure geographical proximity, as shown in Eq. (6).

$$Geoproximity_{mk}=1-\ln(d_{mk}/\max d_{mk})$$ (6)

(2) Institutional proximity. Institutional proximity refers to the similarity that the subjects are restricted by informal institutional constraints (social norms, cultural values) and formal rules (policies, laws and regulations). Institutional similarity decreases uncertainty and reduces transaction costs in cooperation. A strict administrative hierarchy exists in China’s cities, including provincial cities, vice-provincial cities, cities with independent annual plans, principal cities, vice-principal cities, and deputy cities. Similarly, urban policies also have typical administrative hierarchical characteristics. High administrative hierarchical cities generally possess higher marketization of engineering innovation, richer social capital, and more favorable industrial policies. This study utilized the administrative hierarchy of dominant cities to simplify the measurement of institutional proximity.


$$Insproximity_{mk}=\left\{ \begin{array}{cc} & 3\;\text{Cities }m\text{ and }k\text{ are both beyond sub-provincial}\\ & 1\;\text{Cities }m\text{ and }k\text{ have only one beyond sub-provincial }\\ & 0\;\text{Cities }m\text{ and }k\text{ are neither beyond sub-provincial} \end{array}\right.$$ (7)

(3) Cognitive proximity. Cognitive proximity reflects the consistency of cognitive understanding and the similarity of knowledge endowment distribution of cooperation subjects in communication [34]. When two cities are in a similar cultural environment and share similar social norms and values, communication barriers and social trust obstacles are decreased, and cooperation possibilities are increased. Referring to Zhao et al. [38], this study chose whether two cities are in a cultural area to measure cognitive proximity. The value of two cities in the same cultural area is 1; otherwise, it is 0.
(4) Social proximity. Social proximity refers to intimacy and closeness relationships between cooperation subjects. researchers pointed out that social relations accumulating based on friendship, trust and cooperation experience are the catalyst of knowledge spillover. Informal communication has a global promoting effect on the dissemination and learning of tacit knowledge that megaprojects rely on. Participants in network clusters are more inclined to connect with partners of partners. This study chose the overlap degree of two city partners to measure social proximity, calculated by the Jaccard index in Eq. (8).

$$Soc_{mk}=\frac{q_{mk}}{r_{m}+s_{k}-q_{mk}}$$ (8)

Among them, $q_{mk}$ is the copartner numbers of cities m and k; $r_{m}$ and $s_{k}$ are the partner numbers of city m and city k, respectively.

(5)Control variables. Control variables in this study include industry scales and external connectivity. The number of participating enterprises in the cities that won the ZTP was selected to measure industrial scales, and cities’ betweenness centrality was selected to measure external connectivity.

#### 3.4.2 Theoretical model.

The gravity model is a technique mainly utilized to explore the influencing factors of interregional contact [37]. With the purpose of considering the action mechanism of more relation elements on interregional cooperation, Scherngell et al. [35] proposed negative binomial regression of the gravity model. On the basis of [37] and [27], this study constructed a negative binomial regression model to explore the influence mechanism of multidimensional proximity on the CCIN of megaprojects. The regression model is as follows.


$$D_{mk}=\alpha +\sum \beta_{mk}proximity_{mk}+\sum \gamma_{mk}control_{mk}+\varepsilon_{mk}\backslash )$$ (5)

In Eq. (5), dependent variable $D_{mk}$ characterizes the megaproject numbers of cooperation between cities m and k; independent variable $proximity_{mk}$ represents the multidimensional proximity including geographical proximity, institutional proximity, cognitive proximity and social proximity; $control_{mk}$ stands for control variables selected in this study, that is, industrial scales and external connectivity of the innovation cities.

## 4 Spatiotemporal evolution and empirical analysis of megaproject innovation networks

### 4.1 Spatiotemporal evolution and centrality analysis of megaproject innovation networks

Aiming at the CINPU formed by construction units as nodes, UCINET was used to visualize the networks and analyze the networks’ centrality.

#### 4.1.1 Spatiotemporal evolution of the CINPU.

**Fig 1:**
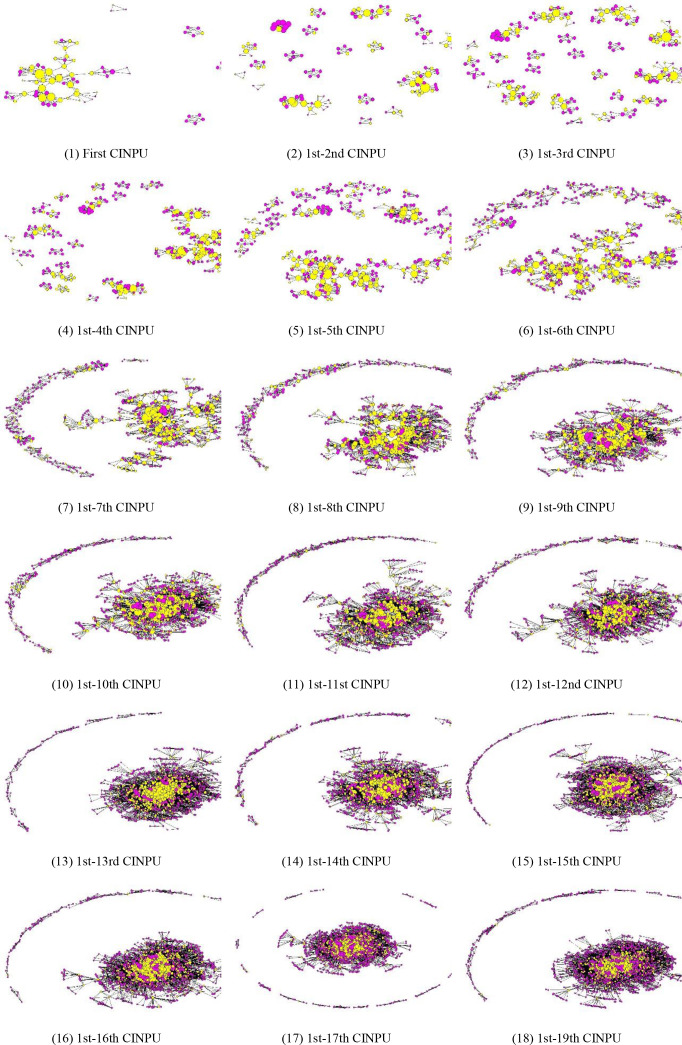
Evolution of the CINPU over time. (1) First CINPU. (2) 1st-2nd CINPU. (3) 1st-3rd CINPU. (4) 1st-4th CINPU. (5) 1st-5th CINPU. (6) 1st-6th CINPU. (7) 1st-7th CINPU. (8) 1st-8th CINPU. (9) 1st-9th CINPU. (10) 1st-10th CINPU. (11) 1st-11st CINPU. (12) 1st-12nd CINPU.(13) 1st-13rd CINPU. (14) 1st-14th CINPU. (15) 1st-15th CINPU. (16) 1st-16th CINPU. (17) 1st-17th CINPU. (18) 1st-19th CINPU.

Fig 1 illustrates the evolution of the CINPU over time. Yellow nodes in Fig 1 represent the participating units that won two or more ZTPs, and pink nodes represent the participating units that only won once ZTP. The node sizes are positively correlated with the degree centrality of the participating units. The following can be seen in Fig 1: (1) The scale of the CINPU continues to expand over time, and the node number in the first CINPU is 78, while the node number in the 1–19th CINPU is 1719. (2) The largest component continues to increase, and the node number in the largest component in the first CINPU is 60, accounting for 76.92%, while the node number in the 1–19th CINPU is 92.79%. (3) The participating units winning two or more ZTP gradually connected the separated small networks into a large-scale connected network and occupied the central position of the networks.

#### 4.1.2 Centrality analysis of the CINPU.

The top 20 participating units in the 1st-19th CINPU are listed in Table 2. Among the top 20 participating units in the degree centrality ranking, the participating units of the railway system occupied 14 places. Among the top 10 participating units in the degree centrality ranking, the participating units of the railway system even occupied 8 places, indicating that the railway system was the key awarded field of the ZTP, and the participating units of the railway system occupied key positions and had absolute power in the networks. Among the top 20 participating units in the degree centrality ranking, 50% of the participating units had won awards in the first three sessions, including 3 in the first session, 1 in the second session, and 6 in the third session. The top 20 participating units had won the ZTP several times; among them, the China Railway Twenty-fourth Engineering Bureau, which had won 8 ZTPs, had the lowest number of awards, and the China Railway Fourth Engineering Bureau, which had won 15 ZTPs, had the highest number of awards. The top 20 participating units had participated in at least 10 megaprojects, of which China Railway Communications and Signaling, with the lowest number of cooperation megaprojects, had participated in 10 megaprojects in all, and China Railway Fourth Engineering Bureau, with the largest number of cooperation megaprojects, had participated in 33 megaprojects in all. The top 20 participating units possessed a large number of cooperation organizations when participating in megaprojects. For instance, the average number of individual project partners of China Railway Communication and Signal Company exceeded 15, indicating that these participating units had strong resource integration capabilities.

**Table 2:** Top 20 participating units in the degree centrality ranking of the 1st-19th CINPU.

Rank	Participating units	Degree centrality	First prize	Total prizes	Total participants	Average number of partners for each megaproject
1	ZTSIJU	313	1	15	33	9.48
2	ZTSANJU	270	1	11	24	11.25
3	ZTSUIDAO	247	3	15	32	7.72
4	ZTERJU	246	7	12	23	10.70
5	ZTYIJU	235	3	11	26	9.04
6	ZTDIANQIHUA	226	4	10	18	12.56
7	ZTSHIERJU	209	6	11	25	8.36
8	BJJIANZHUYUAN	193	3	14	29	6.66
9	ZJBAJU	189	4	14	23	8.22
10	ZTDISIKANCHA	183	1	12	27	6.78
11	ZTSHIBAJU	174	3	12	20	8.70
12	ZTDAQIAOJU	162	3	13	21	7.71
13	ZJDIERHANGWU	161	7	12	21	7.67
14	BJCHENGJIAN	157	6	9	20	7.85
15	ZTERSHISIJU	157	8	8	11	14.27
16	ZTTONGXINXINHAO	152	7	9	10	15.20
17	BJJIANGONG	150	8	9	18	8.33
18	ZTERYUAN	148	2	11	23	6.43
19	BJCHENGJIANSHEJI	143	12	7	14	10.21
20	ZTDISANKANCHA	142	3	11	15	9.47

### 4.2 Network structure and spatiotemporal evolution of the CCIN

This section constructed the CCIN on the basis of the cities where the ZTPs’ participating units are located, introduced UCINET to analyze the core-periphery structure, and utilized ArcGIS to visualize the networks and study networks’ spatiotemporal evolution.

#### 4.2.1 Core-periphery structure analysis of the CCIN.

**Fig 2:**
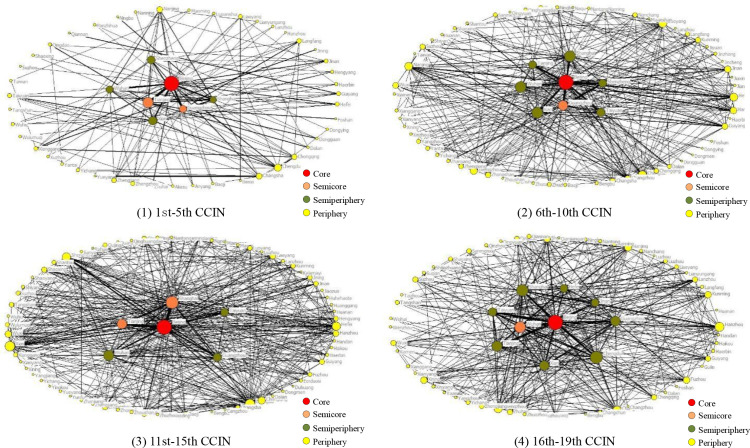
Core-periphery structure of the CCIN. (1) 1st-5th CCIN. (2) 6th-10th CCIN.(3) 11st-15th CCIN. (4) 16th-19th CCIN.

Referring to Liang’ research [37] on the division of core-periphery structure, cities with a core degree ≥ 0.5 were classified as core layers, cities with a core degree between 0.25–0.5 were classified as semicore layers, cities with a core degree between 0.1–0.25 were classified as semiperiphery layers, and cities with a core degree < 0.1 were classified as periphery layers. It can be concluded from Fig 2 that the CCIN presents obvious “core, semicore, semiperiphery, periphery” structures, and the layer structures dynamically evolve over time.

Although Beijing’s core degree increased from 0.778 in the 1st-5th CCIN and then decreased to 0.753 in the 16th-19th CCIN, it was present throughout the core layers of the CCIN. Shanghai was in the semiperiphery layers except for the 11–15th CCIN, while Tianjin and Xi’an shifted between the semicore layers and semiperiphery layers. Guangzhou’s core degree increased slightly from 0.263 in the 1–5th CCIN to 0.265 in the 6–10th CCIN, then decreased to 0.137 in the 11–15th CCIN and 0.196 in the 16–19th CCIN, and fell into the semiperiphery layers from the semicore layers. Shenzhen’s core degree increased from 0.1 in the 1–5th CCIN to 0.164 in the 6–10th CCIN, then decreased to 0.098 in the 11–15th CCIN, continued to decrease to 0.086 in the 16–19th CCIN, and fell into the periphery layers from the semiperiphery layers. Wuhan’s core degree decreased slightly from 0.176 in the 1–5th CCIN to 0.169 in the 6–10th CCIN, then decreased to 0.142 in the 11–15th CCIN, then rose sharply to 0.286 in the 16–19th CCIN, and passed into the semicore layer from the semiperiphery layers. Taiyuan’s core degree rose from 0.029 in the 1–5th CCIN to 0.119 in the 6–10th CCIN, then rose to 0.128 in the 11–15th CCIN, then continued to rise to 0.236 in the 16–19th CCIN, and turned into the periphery layers from the semiperiphery layers. Chengdu’s core degree decreased slightly from 0.088 in the 1st-5th CCIN to 0.076 in the 6th-10th CCIN, rose to 0.080 in the 11th-15th CCIN, continued to rise to 0.231 in the 16th-19th CCIN, and entered the semiperiphery layers from the periphery layers. Jinan’ core degree decreased slightly from 0.05 in the 1st-5th CCIN to 0.046 in the 6–10th CCIN, decreased to 0.034 in the 11–15th CCIN, then rose sharply to 0.12 in the 16–19th CCIN, and changed from the periphery layers into the semiperiphery layers. Hefei’s core degree increased from 0.061 in the 1st-5th CCIN to 0.089 in the 6th-10th CCIN, dropped to 0.057 in the 11th-15th CCIN, then rose to 0.118 in the 16th-19th CCIN, and rose from the semiperiphery layers to the periphery layers.

The core-periphery structure evolution of the CCIN showed that Beijing is a unique city in the core layers, indicating that most of the participating units in the key positions of the CCIN are located in Beijing. The reason for this phenomenon may be that Beijing is the political, economic and cultural center of China, and most corporate headquarters are located in Beijing. In addition to Beijing, Shanghai, Tianjin and Xi’an, the major cities in the CCIN appear to transfer from the eastern first-tier cities, Guangzhou and Shenzhen, to the eastern second-tier cities, Taiyuan, Jinan, Hefei, and the central-western second-tier city Chengdu.

#### 4.2.2 Spatiotemporal evolution of the CCIN.

**Fig 3:**
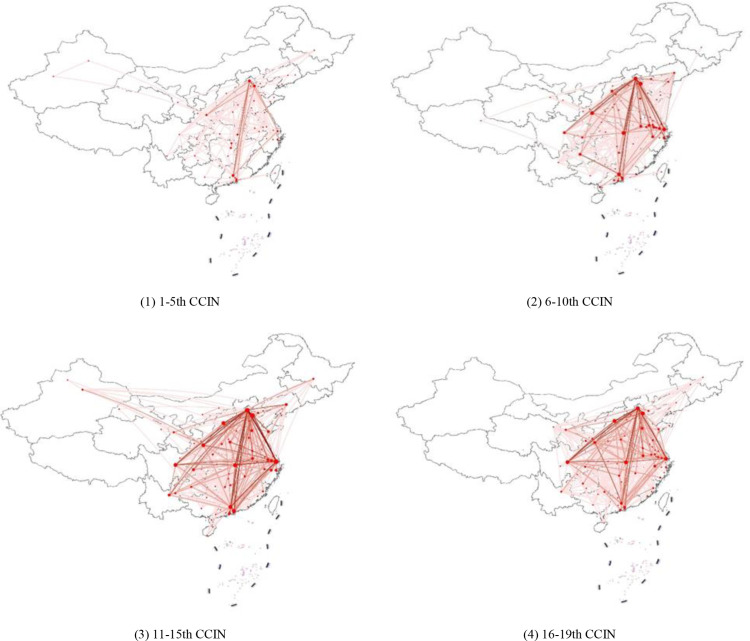
Spatiotemporal evolution of the CCIN. (1) 1-5th CCIN. (2) 6-10th CCIN. (3) 11-15th CCIN. (4) 16-19th CCIN.

With the purpose of further analyzing the spatiotemporal evolution of the CCIN, ArcGIS was introduced to visualize the networks, and Fig 3 exhibits the visualization results. Fig 3 -(1) shows that the cooperation intensity between the cities that won the 1st-5th ZTPs is generally low. The majority of the cooperation is concentrated in Beijing, Tianjin, Wuhan, Guangzhou, Shenzhen, Shanghai and Xi’an. The cities with the highest cooperation intensity are Beijing and Tianjin, Beijing and Guangzhou, Beijing and Wuhan, and Beijing and Xi’an. From Fig 3-(2), it can be seen that the cooperation intensity between the cities that won the 6–10th ZTPs is significantly stronger than those of the 1–5th, and the cooperation intensity between Beijing, Guangzhou, Shanghai, Wuhan and Shenzhen is further enhanced. For instance, the cooperation intensity between Beijing and Guangzhou increased from 20 to 89, and the cooperation intensity between Beijing and Shanghai increased from 14 to 68. In addition, the importance of Tianjin, Xi’an, Taiyuan and Chengdu in the collaborative innovation network was also strengthened. Fig 3 -(3) indicates that the cooperation intensity between cities that won the 11th-15th ZTPs is predominantly higher than that of the 1–5th and 6–10th, and the radiation effect of Beijing as an innovative peak is significantly enhanced. For example, the cooperation intensity between Beijing and Shanghai reached 319, the cooperation intensity between Beijing and Tianjin reached 228, and the cooperation intensity between Beijing and Taiyuan reached 123. The cooperation intensity between the central and western cities of Chengdu, Taiyuan, Xi’an and Wuhan has also been further consolidated; for example, the cooperation intensity between Chengdu and Wuhan increased remarkably from 4 in the 6th-10th to 27. From Fig 3 -(4), it can be concluded that the synergy range of the cities that won the 16th-19th ZTPs obviously contracted to the central, western and eastern coastal cities compared with the 11th-15th, and the cooperation intensity also weakened to a certain extent compared with the 11–15th. The reason may be that, affected by the control of COVID-19 in the past three years, economic activities, commercial exchanges and material flows in the same administrative region were closer and more frequent, and exchanges and cooperation across provincial administrative regions were limited to a certain extent. The partner selections of the participating units were thereby limited by geographical distance, resulting in a narrowing of the scope of collaborative innovation.

### 4.3 Multidimensional proximity mechanism of the CCIN

#### 4.3.1 Correlation Analysis.

This study employed Spass software to conduct a correlation analysis of the variables, with the output of the correlation coefficients for each phase presented in Tables 3–6. The results indicate that in all four phases, the absolute values of the correlation coefficients between the variables are less than 0.6, indicating no issues with multicollinearity.

**Table 3:** Correlation coefficients among variables in the aggregated network of 1-5 sessions.

Variables	$D_{mk}$	$Geo_{ij}$	$Ins_{ij}$	$Gog_{ij}$	$Soc_{ij}$	$Con_{i}$	$Sca_{ij}$	$Con_{j}$
$D_{mk}$	1							
$Geo_{ij}$	0.102	1						
$Ins_{ij}$	0.379**	0.164**	1					
$Gog_{ij}$	0.124*	0.485**	-0.216**	1				
$Soc_{ij}$	0.213**	0.039	0.165**	0.105	1			
$Con_{i}$	0.242**	-0.175**	0.207**	-0.180**	-0.100	1		
$Sca_{ij}$	0.459**	-0.168**	0.399**	-0.265**	-0.094	0.422**	1	
$Con_{j}$	0.242**	-0.175	0.207**	-0.180**	-0.100	-0.232**	0.422**	1

Note: *, **, *** denote significance at the 10%, 5%, and 1% statistical levels, respectively.

**Table 4:** Correlation coefficients among variables in the aggregated network of 6-10 sessions.

Variables	$D_{mk}$	$Geo_{ij}$	$Ins_{ij}$	$Gog_{ij}$	$Soc_{ij}$	$Con_{i}$	$Sca_{ij}$	$Con_{j}$
$D_{mk}$	1							
$Geo_{ij}$	0.035	1						
$Ins_{ij}$	0.328**	-0.188**	1					
$Gog_{ij}$	-0.066	0.053	-0.024	1				
$Soc_{ij}$	0.259**	-0.093*	-0.213**	-0.008	1			
$Con_{i}$	0.327**	-0.143**	0.282**	-0.114**	-0.061	1		
$Sca_{ij}$	-0.044	0.001	-0.136**	-0.205	-0.114	-0.077*	1	
$Con_{j}$	0.293**	-0.143**	0.282**	0.006	-0.069	-0.199**	-0.006	1

**Table 5:** Correlation coefficients among variables in the aggregated network of 11-15 sessions.

Variables	$D_{mk}$	$Geo_{ij}$	$Ins_{ij}$	$Gog_{ij}$	$Soc_{ij}$	$Con_{i}$	$Sca_{ij}$	$Con_{j}$
$D_{mk}$	1							
$Geo_{ij}$	0.024	1						
$Ins_{ij}$	0.348**	0.060	1					
$Gog_{ij}$	0.049	0.519**	-0.165**	1				
$Soc_{ij}$	0.216**	0.040	0.108**	-0.015	1			
$Con_{i}$	0.266**	-0.092**	0.223**	-0.143**	-0.132**	1		
$Sca_{ij}$	0.405**	-0.100**	0.334**	-0.249**	-0.144**	0.527**	1	
$Con_{j}$	0.266**	-0.092**	0.223**	-0.143**	-0.132**	-0.206**	0.508**	1

**Table 6:** Correlation coefficients among variables in the aggregated network of 16-19 sessions.

Variables	$D_{mk}$	$Geo_{ij}$	$Ins_{ij}$	$Gog_{ij}$	$Soc_{ij}$	$Con_{i}$	$Sca_{ij}$	$Con_{j}$
$D_{mk}$	1							
$Geo_{ij}$	0.058	1						
$Ins_{ij}$	0.411**	0.027	1					
$Gog_{ij}$	0.022	0.487**	-0.073*	1				
$Soc_{ij}$	0.348**	-0.115**	0.133**	-0.043	1			
$Con_{i}$	0.330**	-0.059	0.290**	-0.134**	-0.013	1		
$Sca_{ij}$	0.461**	0.017	0.412**	-0.198**	-0.068	0.473**	1	
$Con_{j}$	0.330**	-0.059	0.290**	-0.134**	-0.013	-0.247**	0.473**	1

#### 4.3.2 Regression results.

The collinearity diagnosis between the variables of the 1–5th, 6–10th, 11–15th and 16–19th CCIN was orderly performed, the VIF values of each variable were less than 10, and multicollinearity was thus excluded. The regression results obtained through SPASS are shown in Table 7.

**Table 7:** Regression results of the multidimensional proximity mechanism of the CCIN.

Variables	1st-5th CCIN	6th-10th CCIN	11st-15th CCIN	16th-19th CCIN
$Geo_{ij}$	0.137**	0.200***	0.012	0.079
$Ins_{ij}$	0.208***	0.058*	0.176***	0.119***
$Gog_{ij}$	0.235***	-0.018	0.192***	0.126***
$Soc_{ij}$	0.218***	0.325***	0.293***	0.365***
$Sca_{i}$	0.228***	0.025	0.131**	0.108**
$Sca_{j}$	0.238***	0.026	0.127**	0.118**
$Con_{i}$	0.215***	0.443***	0.259***	0.340***
$Con_{j}$	0.205***	0.416***	0.264***	0.345***
R−square	0.410	0.376	0.341	0.471
Adj R−Sqr	0.393	0.369	0.335	0.466
Data	294	715	910	818

Note: The coefficients in the above table are standardized regression coefficients. *, **, *** are significant at the statistical levels of 10%, 5% and 1%, respectively.

(1) Geographical proximity. The standardized coefficients of geographical proximity in the 1–5th, 6–10th, and 16–19th CNICs were all positive and passed the 1% significance test. This indicates that the closer the geographical distance is, the higher the collaborative innovation intensity between two cities. Megaproject innovation appears to have the characteristics of a temporary cross-layer organizational structure and has the complexities of organizational structure and engineering technology integration. Intense innovation networks containing designers, contractors and other participating units must be built to overcome barriers, and face-to-face consultations and offline agreements on innovation cooperation, technology transfer, and achievement transformation are also crucial to the CCIN. Geographical proximity promotes communication between participating units and the dissemination and diffusion of tacit knowledge and reduces the time and space cost of participating units in the process of contract signing, execution and change. The coefficients of geographical proximity decreased from 0.137 in the 1st-5th CCIN to 0.079 in the 16th-19th CCIN, indicating that the importance of geographical proximity decreased. This may be because, due to the rapid development of communication, information, transportation and other fields, the concepts of agglomeration, 1-hour urban circle and 2-hour urban circle were able to be presented and implied, which greatly improved citizens’ travel efficiency and reduced the negative impact of geographical distance on communications.
(2) Institutional proximity. Institutional proximity passed the significance test in all regressions. Among them, the 10% significance test was successful in the regression of the 6th-10th CCIN, and the 1% significance test was fulfilled in the regression of the other CCINs, indicating that the institutional similarity between cities enhances the innovation cooperation intensity. The government selects the participating units of megaprojects through the bidding system. The megaproject bidding system has typical administrative hierarchical characteristics, including the “Bidding Law of People’s Republic of China” promulgated by the central government, the bidding management regulations issued by provincial governments, and the bidding implementation rules released by provincial capitals and prefecture-level cities. Differences in standards and regulations increase the difficulties of megaproject innovation. Institutional proximity indicates that the related institution is highly similar, and a similar institutional environment is conducive to reducing cooperation uncertainty and eliminating hidden barriers.
(3) Cognitive proximity. In addition to the 6th-10th CCIN, a 1% significance test was passed in the 1st-5th, 11th-15th and 16th-19th CCIN. This indicates that when cities are in a similar cultural environment and share similar social norms and values, the cooperation intensity between them will be stronger. Megaproject innovation is not only subject to the external environment, such as standards, systems, and qualifications but also depends on the dissemination of tacit knowledge and the collaborative communication of innovation subjects. The similarity between the cultural environment and the understanding of cultural values brings status recognition and cultural identity of the same cultural area while reducing the information barriers between the innovation subjects and making the spatial flow of knowledge, information, capital and other elements smoother, thus promoting the collaborative innovation of megaprojects.
(4) Social proximity. The standardized coefficients of social proximity all passed the 1% significance test. Among them, the coefficient was 0.218 in the 1st-5th CCIN, increased to 0.325 in the 6th-10th CCIN, slightly decreased to 0.293 in the 11th-15th CCIN, and increased to 0.365 in the 16th-19th CCIN. In the early stage of megaproject innovation, a large amount of knowledge, capital and manpower are needed, the innovation process is highly complex and deeply uncertain, and the transformation of innovation achievements also has a specific scope. Mutual trust based on good social relations significantly reduces cooperation costs and transaction risks, thereby increasing the possibility of establishing a partnership between two cities. Social proximity represents the overlap degree between two cities’ partners. The higher the social proximity index is, the greater the number of cooperators of two cities, and the higher the cooperation intensity. In other words, cities are more willing to establish cooperative relationships with partners.

#### 4.3.3 Robustness test.

To test the robustness of the models, this study collected the investment amounts of the ZTP projects, substituting the quantity of megaprojects cooperation with the investment amount in cooperation between cities, and conducted regression analysis again. The regression results, as shown in Table 8, confirm the robustness of the theoretical model.

**Table 8:** Robustness test results.

Variables	6th-10th CCIN	11st-15th CCIN	16th-19th CCIN
$Geo_{ij}$	0.072**	-0.040	-0.053*
$Ins_{ij}$	0.092*	0.018	0.010
$Gog_{ij}$	-0.008	0.168***	0.177***
$Soc_{ij}$	0.204***	0439**	0.444***
$Con_{i}$	0.243***	0.440***	0.380***
$Sca_{ij}$	0.015	-0.100**	0.140**
$Con_{j}$	0.233***	0.438***	0.340***
R−square	0.148	0.422	0.471
Adj R−Sqr	0.139	0.416	0.467
Data	678	910	818

Note: *, **, *** are significant at the statistical levels of 10%, 5% and 1%, respectively.

## 5 Conclusions, implications, and limitations

Innovation is an effective way to improve megaproject performance. Megaproject innovation requires collaborative innovation across organizations, departments, industries and regions and gradually forms an innovation network. Existing studies have emphasized the important role of collaborative innovation in megaproject construction, constructed project-based cross-organizational innovation networks, and analyzed the evolution characteristics and topological characteristics of innovation networks. However, there is still a research gap on the network structure, spatiotemporal evolution and multidimensional proximity mechanism of city collaboration networks based on megaprojects. On the basis of the 1st-19th Zhan-Tianyou Prize winning megaprojects, collaborative innovation networks of participating units of megaprojects and city collaborative innovation networks of megaprojects were constructed. Spatiotemporal evolution characteristics and core organizations of the collaborative innovation networks of participating units were analyzed. Core-periphery structures, spatiotemporal evolution characteristics and multidimensional proximity mechanisms of city collaborative innovation networks were discussed. The results show the following.

**Spatiotemporal evolution and centrality analysis of the CINPU.** (1) Scale of the CINPU continues to expand over time. (2) The largest component continued to increase. (3) Participating units winning two or more ZTPs gradually connect the separated small networks into a large-scale connected network and occupy the central position of the networks. (4) Participating units of the railway system occupy key positions in the networks and have absolute power in the networks. (5) Core organizations in the CINPU possess a large number of cooperation organizations, and these core organizations have strong resource integration capabilities.

**Network structure and spatiotemporal evolution of the CCIN.** (1) CCIN presents an obvious “core, semicore, semiperiphery, periphery” structure, and the layer structures evolve dynamically over time. (2) Beijing is the only city in the core layers of the CCIN. (3) CCIN shows a trend of transferring from the eastern first-tier cities Guangzhou and Shenzhen to the eastern second-tier cities Taiyuan, Jinan, Hefei and the central-western second-tier city Chengdu.

**Multidimensional proximity mechanism of the CCIN.** (1) Geographical proximity has a positive effect on the CCIN. However, the standardized coefficient of geographical proximity shows a downward trend, indicating that the importance of geographical proximity has declined due to the rapid development of communication, information, transportation and other fields. (2) Institutional proximity shows a positive effect on CCIN. A similar institutional environment is conducive to reducing cooperation uncertainty and eliminating hidden barriers. (4) Social proximity passed the 1% significance test in all CCINs; that is, cities are more willing to establish cooperative relationships with partners.

The managerial implications of this study are as follows. (1) Vigorously leverage emerging technologies such as artificial intelligence, big data, and the Internet of Things in the field of megaprojects to overcome the limitations of geographical distance on collaborative innovation. (2) When formulating policy documents, enhance communication between local governments and, based on compliance with higher-level laws, increase the coordination of policy documents issued by different cities regarding collaborative innovation in megaprojects. (3) Local governments should strengthen cultural exchanges and scientific and technological cooperation between cities, promote mutual trust and benefit among participating units in major engineering projects, and achieve resource sharing and complementary advantages to enhance collaborative innovation performance.

This study still has some limitations. First, this study only uses a single measure of proximity and does not measure it from multiple perspectives, future research can employ different measurement methods to increase the reliability of the study. Second, this study used the number of megaproject collaborations as the dependent variable, without considering the relationship between network structure and actual innovation outcomes or project performance, future research can include metrics of innovation output (e.g., patents, adopted new technologies) or project performance indicators to link network structure with tangible innovation outcomes.

## Supporting information

S1 data Cooperation 1-5. (XLSX)
